# Antibody Aggregate Removal by Multimodal Chromatography

**DOI:** 10.3390/molecules30112363

**Published:** 2025-05-29

**Authors:** Veronika Rupčíková, Tomáš Molnár, Tomáš Kurák, Milan Polakovič

**Affiliations:** Department of Chemical and Biochemical Engineering, Institute of Chemical and Environmental Engineering, Faculty of Chemical and Food Technology, Slovak University of Technology, Radlinského 9, 812 37 Bratislava, Slovakia; veronika.rupcikova@stuba.sk (V.R.); tomas.molnar1@stuba.sk (T.M.); tomas.kurak@stuba.sk (T.K.)

**Keywords:** antibody aggregates, multimodal chromatography, mAb purification, ion-exchange chromatography, hydrophobic interaction chromatography, aggregate formation

## Abstract

The growing demand for therapeutic monoclonal antibodies (mAbs) has heightened the need for efficient and scalable purification strategies. A major challenge in downstream processing is the removal of antibody aggregates, which can compromise drug safety, efficacy, and regulatory compliance. This review explores the use of multimodal chromatography for aggregate separation, providing an in-depth analysis of commercially available resins and emerging adsorbent prototypes. It also examines the mechanisms of aggregate formation during bioprocessing. A comparative evaluation of conventional single-mode chromatography techniques—affinity, ion exchange, and hydrophobic interaction—is presented alongside multimodal chromatography, which integrates ion-exchange, hydrophobic, and other non-covalent interactions for enhanced aggregate clearance and process flexibility. The review primarily assesses commercial multimodal resins in terms of aggregate removal efficiency, binding capacity, and scalability. Additionally, advancements in prototype resins and multimodal membranes are discussed. Finally, the advantages, limitations, and future directions of multimodal chromatography in mAb aggregate removal are outlined. As purification demands continue to evolve, multimodal chromatography is poised to play an increasingly critical role in achieving the high purity standards required for therapeutic antibodies.

## 1. Introduction

The first application of antibodies as therapeutics dates back to 1890 when Kitasato and Behring used serum from immunized animals to cure tetanus and diphtheria [1]. Since then, antibodies and technologies for their production have undergone intensive progress, becoming the dominant group among therapeutics. According to the Antibody Society, there were 136 immunoglobulins in the late stages of clinical trials as of 1 November 2023 [2]. In 2018, the value of the global antibody market was USD 115.2 billion, and by 2025, its estimated value is projected to reach USD 300 billion [3].

Despite the natural presence of antibodies in the organism, their irreplaceable character in clinical and research applications is a driving force for the creation of reliable and repeatable methods of their production. The most common method for the production of therapeutic mAbs is the expression by recombinant mammalian cell cultures. The use of recombinant cells is highly efficient for large-scale manufacturing due to the rapid growth and stability of the cells [4,5]. The most commonly used cells for mAb production are Chinese hamster ovary (CHO) cells or human embryonic kidney 293 (HEK293) cells [5]. According to Dhara et al., CHO cells account for the production of two thirds of the approved therapeutic mAbs in the world [6].

Cell culture media contain various impurities, including aggregates of the target protein, which pose health hazards when introduced to patients. Therefore, a high purity of the preparations is necessary. This is achieved in a sequence of downstream processing operations, which typically include centrifugation, filtration, low-pH virus deactivation, and several chromatography steps. The downstream processing operations form the majority of production expenses.

This review article provides a summary of existing chromatography methods used in antibody polishing, with special emphasis on the removal of aggregates. The introductory part of this review is devoted to antibody aggregates as a crucial undesirable element of culture broth, their characterization, and the causes of their formation. It is followed by a summary of classical chromatographic methods commonly used for antibody polishing, mentioning studies that explore different options for improving these methods. The essential part of this work addresses the use of mixed-mode or multimodal chromatography for the separation of antibody aggregates. It includes an overview of the most commonly used mixed-mode resins, as well as newly developed resins and ligands. This part includes a section devoted to the special type of mixed-mode chromatography—hydrophobic charge induction chromatography—which appears to be a promising rival to affinity chromatography for capturing antibodies from culture broth. One section also focuses on the use of mixed-mode adsorbents in the form of membranes, which could help to reduce downstream costs.

## 2. Aggregate Formation

Immunoglobulins (Igs) are glycoproteins with a characteristic Y-shaped molecule and molecular weight of 150 kDa. Ig monomers consist of two heavy and two light peptide chains, which are connected by a flexible sulphide bond in the hinge region. Each peptide chain has an NH2 terminus, forming part of the variable domain. These variable domains, located at the tips of the Y-shaped arms, are responsible for antigen binding. The stem of the Y-shaped molecule is known as the constant region, which is crucial for binding antibodies to B lymphocytes [7]. Based on the composition of the constant region, five classes of immunoglobulins are distinguished: IgA, IgD, IgE, IgG, and IgM. The most common mammalian antibody is IgG, which is found in blood and tissue fluid.

Antibodies are industrially produced in two forms: polyclonal and monoclonal. Polyclonal antibodies (pAbs) are naturally produced in organisms as part of the immune response to antigen irritation. An antigen typically has several epitopes—specific sites where antibodies can bind. Polyclonal antibodies are a complex mixture, with each antibody molecule specific to a different epitope on the antigen. This diversity makes pAbs suitable for a wide range of applications [8]. Polyclonal antibodies are commonly produced by immunizing animals with a specific antigen. In contrast, monoclonal antibodies (mAbs) are identical in their peptide sequence, as they are produced by cloned B lymphocytes derived from a single parent cell. Each mAb molecule binds to the same epitope on the antigen, providing high specificity in targeting [9].

Commercial mAbs are primarily produced using recombinant mammalian cell lines. During production and downstream processes, mAbs are subjected to rapid changes in physical conditions, which can negatively impact their stability [10]. The aggregation of mAbs is of significant concern, as it can affect the quality of the pharmaceutical product and may also lead to immunogenic responses or renal failure [11,12]. To mitigate health risks, minimizing the amount of aggregates in therapeutic formulations is a crucial requirement that must be met.

**Figure 1:**
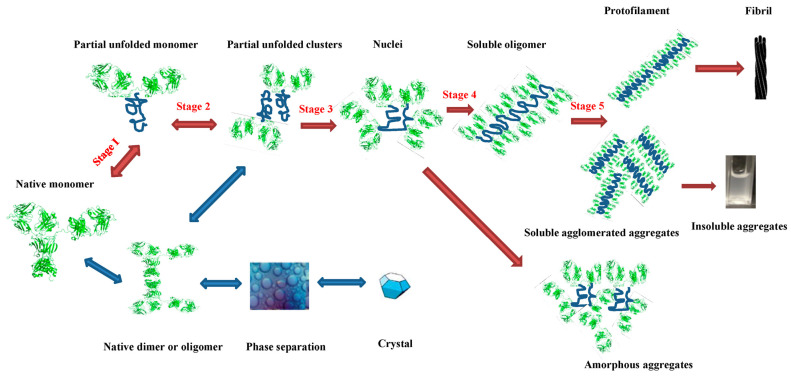
Schematic representation of the antibody aggregation process. The red arrows represent the non-native aggregation, while the dark blue arrows denote the native aggregation. The bidirectional arrows show the reversible steps, and the mono-directional arrows account for the irreversible process [11].

The propensity for mAbs to form aggregates is influenced by both their structural characteristic and the surrounding environmental conditions. The aggregation process involves multiple steps, beginning with partially unfolded monomers associating into oligomers [11]. This process is illustrated in Figure 1. Short aggregation-prone regions (APRs), coupled with low net charge and a high propensity for β-sheet formation, are key factors in aggregate formation. Under normal conditions, these APRs are shielded within the hydrophobic core of the protein, reducing the risk of aggregation [13]. However, increased exposure of hydrophobic groups, particularly at the gas/liquid interface where the air’s hydrophobicity maximizes this exposure, can lead to more pronounced aggregate formation [14].

The pH level significantly affects the net charge and colloidal stability of mAbs [12,15]. Additionally, there is a correlation between higher salt concentrations and an increased content of secondary β-sheet structures in mAbs [16,17]. Typically, weak noncovalent interactions between unfolded monomers lead to reversible aggregation, which can be mitigated by reducing protein concentration or adjusting pH or ionic strength [12,17,18,19,20].

The impact of these conditions can also be moderated by the choice of buffer or the addition of stabilizers [12,16]. Temperature changes can significantly influence the nature of aggregates; for example, under normal storage conditions (2–8 °C) and at lower pH, reversible dimers are predominant. There is an inversed pH dependence for high temperatures. However, at elevated temperature (37 °C), these dimers can aggregate into higher-order structures. Clip-mediated aggregation, which leads to irreversible aggregates, can also occur [17,21,22], often facilitated by the formation of disulphide bonds between cysteine residues [19,20].

Aggregation can also be influenced by different surfaces. For instance, when antibodies adsorb onto stainless steel surfaces, hidden residues are exposed, leading to oxidation of these groups and subsequent aggregation [23]. Similarly, IgG aggregation at the water–silicon oxide interface was studied [24]. Aggregation can also occur during adsorption on the surface of chromatographic resins where the resin structure plays a significant role. Chromatographic resins with tentacle-like structures destabilise the protein, leading to aggregation. During desorption, some of unfolded intermediates refold into their native monomeric form, while others form aggregates. This process can be mitigated by high protein concentrations and carefully selecting operating conditions [25,26,27].

Gillespie et al. observed an unexpected two-peak chromatographic profile for an aglycosylated mAb [28]. After size-exclusion chromatography analysis, they detected an increase in aggregates following elution from a cation-exchange (CEX) resin. Fourier transform infrared spectroscopy and hydrogen–deuterium exchange (HX) experiments revealed that protein molecules undergo denaturation upon adsorption to the resin surface. To prevent aggregate formation, they tested various conditions and stabilisers, finding that citrate and arginine were the most effective.

Building on this work, Guo et al. investigated the unfolding and aggregation of an aglycosylated antibody during adsorption on a strong CEX resin. They found that aggregate formation increased with stronger binding (low salt, low pH) and proposed a conceptual model to explain this phenomenon [26]. Guo and Carta further used HX-MS to explore the mechanisms underlying aggregation during adsorption, confirming their hypothetic model [29]. Huang et al. extended this research on three cation-exchange resins with varying hydrophobicity [30]. Differential scanning calorimetry measurements showed that antibody binding to CEX resins negatively affected structural stability, with more hydrophobic resins having a greater destabilising effect on bound antibodies.

The phenomenon of aggregate formation during adsorption has also been studied for multimodal resins. Roberts and Carta observed an increase in aggregate formation during the elution phase [31]. This aggregation occurred only if no NaCl was present in the column loading buffer, leading to stronger protein binding on the adsorbent [31]. The second part of their study was focused on investigating the mechanism of aggregate formation and the conditions influencing this process [32]. They found that lower salt concentrations and higher pH levels reduce the stability of monomers, leading to aggregation of the bound molecules. Their findings suggest that the hydrophobicity of ligands plays a significant role in promoting aggregate formation, which aligns with the work by Huang et al. Furthermore, protein association in multimodal adsorbents is a key focus in the research conducted by Muca et al. [33].

## 3. Aggregate Removal Using Single-Mode Chromatography

The post-culture medium in mAb production is a complex mixture that contains target proteins alongside product-related impurities such as high-molecular weight (HMW) aggregates, and process-related impurities, including DNA, endotoxins, viruses, and host cell proteins (HCP). Achieving the required purity for immunoglobulins involves multiple separation steps. Pioneering contributions from companies like Amgen, Genentech, and Biogen have shaped the development of platform separation processes for antibodies [34].

A typical downstream cascade in mAb production typically begins with the removal of cells and cell debris, which is usually performed through centrifugation followed by depth filtration. The clarified liquid fraction of the culture broth is then subsequently processed by protein A chromatography, a highly selective technique for capturing immunoglobulins from the complex mixture of dissolved compounds. Protein A adsorbents yield an intermediate mAb product with relatively high purity. However, this intermediate still contains impurities such as residual DNA; product-related impurities, such as aggregates and low molecular weight species; HCP; and leaked protein A. Notably, the low pH used in the elution phase of protein A chromatography can induce aggregate formation.

Further removal of these undesired components, ensuring the safety and efficacy of the final preparation is accomplished through several chromatographic polishing steps, typically preceded by viral inactivation and filtration. Amgen’s platform employs cation-exchange (CEX), anion-exchange (AEX), hydrophobic interaction, and hydroxyapatite chromatography [35]. Genentech has adopted a streamlined two-column polishing strategy, utilizing AEX in flowthrough mode for HCP removal and CEX in bind-elute mode for the removal of HMW aggregates and residual HCP [36]. Additionally, operating AEX in weak partitioning mode has been shown to reduce the need for CEX in downstream processing [37].

Despite the dominant role of chromatography in polishing steps, alternative methods for aggregate removal have also been explored. For example, Wan et al. employed diafiltration to separate monoclonal antibody monomers from dimers and oligomers [38]. McDonald et al. investigated the removal of impurities, including antibody aggregates, through precipitation induced by the addition of anionic polyelectrolytes [39]. Both studies highlighted the critical importance of optimizing pH, ionic strength, and other process conditions to achieve effective separation.

Recent trends in antibody purification have been summarized in several comprehensive reviews [40,41,42]. Gagnon, for example, explored strategies for preventing aggregation and restoring monomeric forms [40]. Vázquez-Rey and Lang provided a detailed overview of aggregation mechanisms during industrial mAb production, focusing on unit operations that may induce aggregation and offering practical recommendations to mitigate this phenomenon [12].

### 3.1. Affinity Chromatography

As mentioned above, affinity chromatography is a key capture step in antibody downstream processing due to its high selectivity [43,44]. The most commonly used ligand for this purpose is protein A (ProA), originally isolated from the bacterial cell wall and now produced recombinantly in *E. coli* cells [45]. ProA resins vary in matrix type, pore size, particle size, and ligand coupling method, all of which influence separation performance.

Hahn et al. compared 15 different commercial ProA resins, reporting static binding capacities of 55–67 mg/mL for agarose-based media and approximately 40 mg/mL for others [46]. The highest dynamic binding capacity, approximately 35 mg/mL, was achieved using MabSelect resin. UltraLink Protein A, UltraLink Protein A Plus, and Protein A Toyopearl exhibited very strong IgG binding. For elution, 0.1 M glycine-HCl pH 3.5 buffer was used, with regeneration carried out using the same buffer at pH 2.5. Even under these harsh conditions, certain resins, like Toyopearl, exhibited strong IgG binding and stability [46].

It is common practice to use eluents such as 20 mM HCl, 0.1 M sodium citrate at pH 2.5, 1 M propionic acid, or a 0.1 M mixture of glycine-HCl at pH 2.0 [47]. However, the use of such harsh conditions to dissociate strongly bound antibodies can lead to aggregate formation. Optimizing elution conditions is therefore a critical task in this type of chromatography.

Arakawa et al. tested various elution buffers to address this issue [48]. Using a conventional 0.1 M citrate buffer at pH 2.9, they observed conformational changes in IgG molecules, resulting in aggregates constituting up to 40% of the protein content. By switching to a buffer containing 0.7 M arginine at pH 4, the recovery degree exceeded 90%, with aggregate formation reduced to less than 1%. Another tested alternative involved increasing the citrate buffer concentration to 2 M at pH 4.3. However, this approach did not yield the desired results, as the highest recovery degree was achieved at a buffer concentration of 0.5 M. Even then, it was less than 30%, significantly lower than the recovery degree obtained with arginine-based elution [48].

A different approach for aggregate removal has been described by Bian and Holstein [49]. This work introduces new ProA resins based on the C domain of protein A. Using gradient or stepwise pH elution, at least 30% of the aggregates were eluted prior to the target protein, with the additional separation of aggregates occurring after the target protein was eluted [49].

Another challenge associated with ProA chromatography is resin fouling caused by nonspecific interactions. Sodium hydroxide is commonly used for resin sanitation, but ProA exhibits low tolerance to highly alkaline environments, leading to ligand leakage and a reduced resin lifetime [50]. To address this limitation, various modifications of ProA have been explored to enhance its resistance to alkaline conditions and to improve the stability of affinity adsorbents [51,52]. Wienberg et al. have demonstrated that modifying the ligand with polyethylene glycol significantly reduced nonspecific interactions with BSA aggregates, thereby increasing its robustness [52].

A biomimetic peptide ligand, designed based on the affinity motif of ProA, has demonstrated effective binding of both mAb aggregates and monomeric mAbs [53,54]. In purification processes utilizing this affinity adsorbent for various feedstocks, it was found to be beneficial to remove HCP using AEX prior to affinity chromatography, as this step helps to prevent HCP-induced aggregation during the process [54].

Although most affinity adsorbents are resin-based bead particles, membranes also serve as excellent supports for affinity ligands. They offer several advantages, including high flow velocities, low pressure drops, convective mass transport, and rapid processing times. Denizli and Arica demonstrated a ProA membrane based on polyhydroxyethylmethacrylate support with a high dynamic binding capacity for IgG, efficient antibody elution using 0.1 M aminoacetic acid, and effective membrane reusability [55]. Similarly, Gehrmann et al. investigated the potential of three ProA membrane adsorbers for mAb purification from cell culture supernatants, reporting volumetric productivities several times higher than those achieved with bead adsorbents [56].

### 3.2. Ion-Exchange Chromatography

Ion-exchange chromatography (IEX) is a key component of the platform processes used in antibody purification. As previously mentioned, IEX is extensively employed in the polishing stage of antibody production. The principle of IEX involves the attraction between a protein and a ligand carrying opposite charges. One of the most significant works elucidating the mechanism of protein separation by IEX is the monograph of Yamamoto et.al [57]. Additionally, numerous chapters in scientific journals and books discuss the methods and applications of ion-exchange chromatography in antibody purification [58,59,60,61].

A comprehensive review focusing on the application of IEX on the characterization of therapeutical proteins is provided by Fekete et al. [62] Another noteworthy review of Yigzaw et al. examines the use of both CEX and AEX chromatographic media for the clearance of mAb aggregates [63]. This review also discusses the optimization of critical separation factors, including binding and elution conditions, column loading, and peak collection. A detailed study by Fekete et al. focused on the characterization of several CEX resins by evaluating their performance in separating different mAbs [64]. This work highlights the importance of resin selection and performance testing in optimizing IEX processes.

The optimization and the improvement of IEX chromatography for antibody separation have been the focuses of many research papers. Ishihara et al. investigated the optimization of linear gradient and stepwise elution applied to CEX purification of mAb with HiTrap SP Sepharose FF [65]. Suda et al. compared common and dextran-grafted Q and SP Sepharose adsorbents for the separation of BSA and IgG aggregates from their monomeric forms [66]. They observed the stronger binding of aggregates to ion-exchangers when compared with monomers, and no differences in purity and yield were detected for IgG when using grafted versus non-grafted SP Sepharose.

The removal of antibody aggregates by IEX chromatography is the central theme of a comprehensive study by Stone et al., where Eshmuno CP-FT was tested in frontal mode and compared with six other commercial CEX resins [67]. Quality by design principles were implemented in the work of Xu et al. to develop a robust process for aggregate separation [68]. The study began with screening six CEX resins, followed by the optimization of elution conditions before concluding with an evaluation of the effects of operating parameter changes with respect to aggregate removal were evaluated.

Kluters et al. aimed to enhance the effectivity of aggregate separation using CEX resins by testing mobile phase modulators, including different polyethylene glycols (PEGs) [69]. To prevent protein precipitation at higher PEG concentrations, the addition of amino acids and polyols to the mobile phase was successfully tested.

Thanks to advances in recombinant technologies, the upstream phase of mAb manufacturing has made steady progress, resulting in higher product titers. However, as product titers increase, so do the levels of impurities and by-products, posing a challenge for the downstream process. When product concentrations reach 5 g/L, 91% of specific manufacturing costs are attributed to downstream processing [70]. As upstream efficiency improves and the industry shifts toward continuous technologies, membrane chromatography has emerged as a potential solution to enhance downstream capacities and productivity. This topic is discussed in several recent reviews [71,72].

Knudsen et al. explored the use of IEX membranes for purifying antibodies from host cell proteins at the process scale [73]. Madadkar et al. focused on using a CEX membrane Sartobind S to purify antibodies from aggregates [74]. Their study involved testing three different bed volumes, ranging from 1 to nearly 10 mL, for the separation of three types of antibodies.

Another promising alternative to traditional packed-bed chromatography is fibre-based adsorbents. Schwellenbach et al. prepared strong CEX fibres and evaluated their effectiveness for separating four model proteins [75]. Winderl et al. applied fibre-based CEX adsorbents for the separation of mAb aggregates [76].

### 3.3. Hydrophobic Chromatography

Hydrophobic chromatography (HIC) is commonly employed as a supplementary step in antibody polishing. Separation in HIC is based on differences in hydrophobicity between substances. In practice, HIC media have lower ligand densities than reverse-phase chromatography, requiring milder elution conditions. Consequently, the risk of protein denaturation and precipitation are reduced [77]. Typically, alkyl and aryl groups are used as hydrophobic ligands.

**Figure 2:**
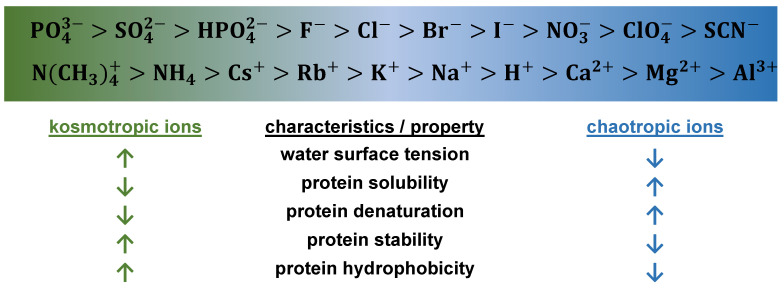
Hofmeister series of anions and cations and their influence on protein solution properties. Upward (↑) and downward (↓) arrows indicate an increase or decrease, respectively, of specific protein property [83].

Adsorption of proteins onto HIC resins is driven by salt concentration, and the influence of salt ions on hydrophobic interactions is explained by the Hofmeister series. Figure 2 illustrates the arrangement of salt ions in the Hofmeister series and their effects on proteins. Kosmotropic or anti-chaotropic salts present in the mobile phase, such as ammonium sulphate, enhance hydrogen bonding in water and increase its surface tension, promoting the hydrophobic interactions of proteins [78,79,80]. Several studies summarize the fundamental principles and factors affecting the HIC of proteins [79,81,82].

A comprehensive review by Fekete et al. discusses the use of HIC for characterizing mAbs and antibody–drug conjugates [84]. The review also covers the impact of chromatographic conditions on antibody adsorption behaviour. Baca et al. investigated the effects of mobile phase composition on the retention of several proteins, identifying salt type and concentration as the most influential factors [85]. However, some studies focus on developing HIC methods for antibodies that do not rely on salt-based hydrophobic interactions [86,87].

Research has also focused on modulating the mobile phase and additives to improve antibody separation. Rodrigues-Aller et al. conducted a detailed study employing various approaches, including gradient modification, pH variation, salt concentration adjustments, and the addition of organic modifiers [88]. In Part 2 of this article series, the authors tested different stationary phases and salts in the mobile phase [89].

Specific efforts have been made to enhance the HIC-based separation of antibody aggregates. A review by Lu et al. summarizes numerous industrial-scale improvements applied to HIC for aggregate separation [90]. McCue et al. investigated the effect of ligand density on HIC resins, focusing on the separation of aggregates from monomeric antibodies [91]. Bresolin et al. achieved more than 99% purity in recombinant antibody polishing using Toyopearl Phenyl 650M [92]. Hall et al. optimized aggregate and HCP separation by using hexylene glycol and arginine as mobile phase additives [93].

The recent trend in cancer therapeutics has seen an increasing reliance on monoclonal antibodies (mAbs) conjugated to cytotoxic payloads, forming antibody-drug conjugates (ADCs). These payloads are often highly hydrophobic, which significantly increases the overall hydrophobicity of the ADC molecule. This characteristic enhances the risk of aggregation, a critical quality and efficacy concern during both conjugation and downstream processing. Aggregation not only reduces therapeutic efficacy but also raises the risk of immunogenicity [94,95]. Subtle differences in the physicochemical properties of over-conjugated DARs and aggregates can be effectively used to remove these species. The increased hydrophobicity of aggregates leads to stronger retention on HIC columns, enabling efficient resolution [96,97].

HIC has also been implemented in membrane formats. Studies by Yoo et al. and Ebert and Fischer-Frühhloz highlight the application of HIC membranes for separating mAbs from impurities [98,99]. A comprehensive process methodology incorporating HIC for antibody purification is detailed by Azevedo et al. [100]

## 4. Aggregate Removal Using Multimodal Chromatography

### 4.1. Multimodal Chromatography: Basic Concepts

In recent decades, a promising alternative to traditional chromatography methods has emerged—multimodal or mixed-mode chromatography. Multimodal adsorbents provide more various types of interaction between proteins and the stationary phase, offering improved selectivity. The most common interactions involve ion exchange, hydrogen bonding, and hydrophobic interactions. The relative strength and prevalence of each interaction depend on the experimental conditions. As mentioned previously, salt type and concentration, and also pH, each have an effect on ion and hydrophobic interactions. Thus, method development and optimization are complex tasks [101]. Mixed-mode chromatography is most commonly utilized in a bind-elute mode [102]. Hydrophobic interactions are typically mediated by aromatic or aliphatic groups, while ion-exchange interactions are mainly driven by amino, carboxyl, and sulfonic groups [103].

Protein aggregates often exhibit increased hydrophobicity and altered charge distributions compared with monomers, resulting in stronger interactions with the hydrophobic regions of multimodal chromatographic ligands. This behaviour has been observed during aggregate/monomer separation in frontal chromatography, where bound monomers were displaced by aggregates, leading to monomer peak overshoot. Under inappropriately chosen conditions (low salt concentrations), bounded monomers can gradually convert to higher-order oligomers that are no longer effectively replaced by aggregates in the feed, ultimately leading to poor separation and reduced monomer yield [32].

As noted earlier, cultivation media are complex mixtures containing numerous impurities. The presence of multiple types of interactions within a single adsorbent allows for the efficient separation of various impurities in a single step, thus increasing productivity. A key challenge in traditional multi-step chromatography is the need to alter conditions between individual columns. For example, cell culture media and eluates from ProA chromatography often contain high salt concentrations, which must be removed due to their negative impact on the performance of IEX chromatography. This usually requires the installation of additional devices, such as inline filters. In contrast, multimodal resins, owing to their ability to utilize hydrophobic interactions, are considered salt tolerant. This characteristic can eliminate the need for desalination steps in downstream processing [104]. For this reason, product fractions from ProA chromatography are commonly used as feed material for multimodal chromatography columns.

Beyond their tolerance for higher salt concentrations in comparison to traditional IEX resins and wide pH range, multimodal adsorbents offer additional benefits. These resins typically have a higher loading capacity—10–100 times greater than reverse-phase chromatography. By adjusting the separation conditions, one can preferentially enhance specific types of interaction, making multimodal resins a flexible and valuable tool for protein separation [105,106,107,108].

The fundamental principle behind the preparation of mixed-mode stationary phases involves attaching various functional groups to the matrix via chemical bounds. Unlike traditional chromatography, where the ligand has a single active functional group, mixed-mode resins feature several functional groups that allow proteins to bind through different types of interactions. The preparation of multimodal resins can follow several strategies [109], including directly coupling different ligands to the matrix, although this may result in inhomogeneous adsorption media, linking separate ligands to a scaffold to define a 3D structure, or using materials that change their properties in response to environmental conditions, such as temperature or pH.

To facilitate ligand accessibility, spacer arms are often used when connecting ligands to the matrix, ensuring that the ligands remain exposed without interfering with protein binding. These spacer arms must be carefully chosen to avoid interfering with the proteins [110]. The matrix material for chromatography media is typically composed of silica gel, cross-linked agarose, or various polymers. However, secondary interactions between these compounds and the solutes can lead to lower selectivity, as undesired compounds may bind nonspecifically. Additionally, these secondary interactions can cause steric hindrance, reducing binding capacity [111,112]. To mitigate these effects, end-capping (blocking free matrix functional groups) or selecting appropriate mobile phases can be employed [111].

Temperature is a critical parameter influencing both the binding thermodynamics and aggregation behaviour of proteins during multimodal chromatography (MMC). Multiple studies have demonstrated that temperature can modulate protein–resin interactions in complex and resin-specific ways, with implications for both separation efficiency and product stability. This feature is salt dependent. At low ionic strength, increasing temperature generally leads to decreased retention, consistent with the weakening of electrostatic interactions. While at high salt concentrations, where hydrophobic interactions dominate, retention increases with temperature suggesting enhanced hydrophobic binding due to the desolvation of non-polar groups. Different MM resins offer unique thermodynamic profiles due to variations in ligand density and ligand structure, influencing their temperature response [33,113,114]. Moreover, while higher temperatures can enhance resolution and selectivity, they also increase the risk of aggregation [17,32].

The concept of mixed-mode resins was first introduced in the 1950s by Tiselius, who used a hydroxyapatite-packed column for protein separation [115]. Since then, the interest in mixed-mode chromatography has grown rapidly, particularly in the biopharmaceutical industry for protein separation applications [116]. This section focuses on commercially available multimodal adsorbents and their applications in the removal of antibody aggregates. Mixed-mode resins are predominantly provided in bead form. While most resins rely on ion-exchange mechanisms as their primary mode of interaction, others primarily utilize metal affinity or hydrophobic interactions.

### 4.2. Commercially Available Multimodal Adsorbents

#### 4.2.1. Hydroxyapatite Resins

Hydroxyapatite (HA), a mineral composed of calcium and phosphate with the formula (Ca_5_(PO_4_)_3_OH)_2_, is historically the oldest type of mixed-mode adsorbent used in the chromatographic separation of proteins, dating back to the 1950s [117]. The Web of Science database lists more than 2500 references to applications involving hydroxyapatite chromatography. Although the term “hydroxyapatite chromatography” first appeared in the 1960s, it gained recognition as one of the principal types of chromatography in the 1970s. However, the frequency of this term’s use has declined over the past 25 years, as hydroxyapatite has come to be recognized as a subset of multimodal adsorbents.

**Figure 3:**
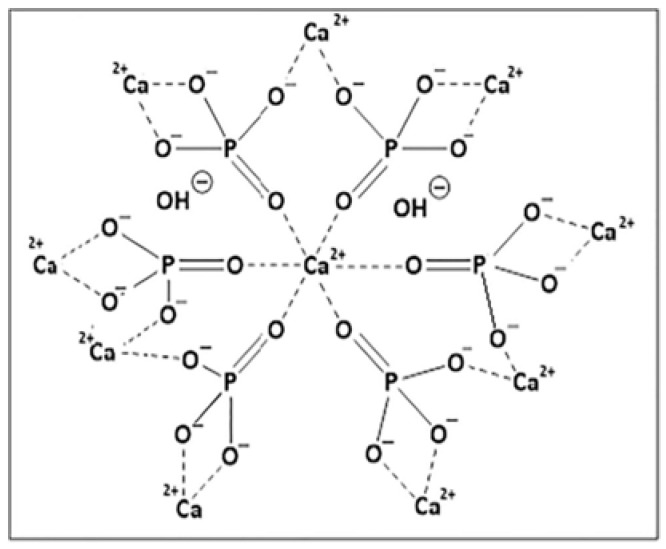
The structure of hydroxyapatite.

Bio-Rad (Hercules, CA, USA) is the primary producer of hydroxyapatite and modified hydroxyapatite chromatographic resins. Their product portfolio includes ceramic resins formed by hydroxyapatite (CHT Type I, CHT Type II, and CHT XT), fluoroapatite (CFT), and hydroxyfluoroapatite (MPC) and hydroxyapatite gel resins (Bio-Gel HT and Biogel HTP). Among these, the most widely used are the ceramic hydroxyapatite resins: CHT type I, which has a higher capacity for binding acidic proteins and CHT type II, which is employed for the separation of albumin and various classes of immunoglobulins [118,119,120]. The molecular structure of hydroxyapatite is depicted in Figure 3. In fluoroapatite, OH- ions are substituted with fluoride ions, F-.

These materials have found applications across a broad range of processes due to their ability to form coordination bonds and facilitate weak anion exchange via Ca^2+^, as well as cation exchange mediated by phosphate groups. Notably, coordination bonds are almost entirely independent of conductivity. Protein binding typically occurs in a phosphate buffer of low ionic strength, with elution achieved using NaCl or a phosphate gradient of increasing ionic strength [121]. Characterization of these two adsorbents and evaluation of competitive binding within a mAb monomer–dimer mixture is detailed in work by Wang and Carta [122]. The results of this study, which prove a better suitability of CHT type II for the removal of higher antibody structures, are summarised in Table 1.

A specific type of hydroxyapatite-based resin is HA Ultrogel, produced by Sartorius (Göttingen, Germany). This resin features microcrystalline hydroxyapatite embedded within a cross-linked agarose matrix and has been manufactured since the 1970s. Despite its long production history, relatively few applications of this resin have been reported in the scientific literature, with most focusing on enzyme purification.

The strength of interactions between proteins, impurities, and the stationary phase in mixed-mode adsorbents can be modulated by adding modifiers to the mobile phase. For instance, Gagnon has demonstrated the use of CHT types I and II for separating IgG and IgM from aggregates in the presence of polyethylene glycol (PEG) [123]. Increasing PEG concentration in the mobile phase improved aggregate separation. At 5.6% PEG in a phosphate gradient, baseline separation of aggregates from monoclonal IgG and IgM was achieved. When a NaCl gradient was used, baseline separation required only 3.75% PEG. Further insights into this strategy are provided in a collaborative study by Gagnon and Beam [124].

Comparative evaluations of various mixed-mode adsorbents for separating antibody aggregates are common. In one such study, Chen et al. compared four types of hydroxy-/fluoroapatite resins and two anion-exchange based mixed-mode resins [125]. CHT type I exhibited the best performance in reducing dimers and HMW aggregates. However, the multimodal anion exchanger MEP Hypercel, produced by Sartorius (Göttingen, Germany), achieved the most effective overall removal of HMW aggregates when hydrophobic interactions were enhanced by lowering buffer conductivity during elution.

In common multimodal adsorbents, hydrogen bonds are supplementary interactions. However, a few years ago, CIMmultus™ H-Bond™ ADC was invented. This multimodal monolith resin features strong hydrogen-donor ligands grafted to the hydrogen-acceptor matrix, establishing hydrogen bonding as the predominant interactions [126]. This type of resin is currently used for separation of large particles such as aggregates, viruses and DNA [127,128].

**Table 1:** The most commonly used hydroxyapatite and multimodal AEX resins and selected applications.

Adsorbent	Operational Mode	Position	Product	Reference
CHT type I	Batch	Polishing step	Monomeric IgG (aggregates selectivity 4.3)	[122]
CHT type II	Batch	Polishing step	Monomeric IgG (aggregates selectivity 5.8)	[122]
MEP HyperCel	Bind-elute	Capture step	mAbs with yield in interval 89–96%	[129]
Capto Adhere	Bind-elute	Polishing step	Monomeric IgG with 92% recovery	[130]
Nuvia aPrime 4A	Batch	Polishing step	Monomeric mAb with >92% purity	[131]
HEA HyperCel	Flowthrough	Post CEX	Monomeric mAb with 98.8% purity and 92% recovery	[132]
PPA HyperCel	Bind-elute	Capture step	mAb with 93% yield	[133]

#### 4.2.2. Multimodal Ion Exchangers

The largest group of commercially available multimodal adsorbents consists of multimodal ion exchangers. These adsorbents typically contain ligands with functional groups that enable both ion-exchange and hydrophobic interactions. Like their single-mode counterparts, mixed-mode ion exchangers contain similar ionizable functional groups and can be classified as strong or weak ion exchangers, depending on the nature of their ionic functional groups. However, in mixed-mode ion exchangers, non-ionic interactions become more prominent when ion-exchange mechanisms are suppressed. This effect is particularly pronounced in weak multimodal ion exchangers, where ion-exchange interactions are more easily diminished under specific conditions.

##### Multimodal Anion Exchangers

**Figure 4:**
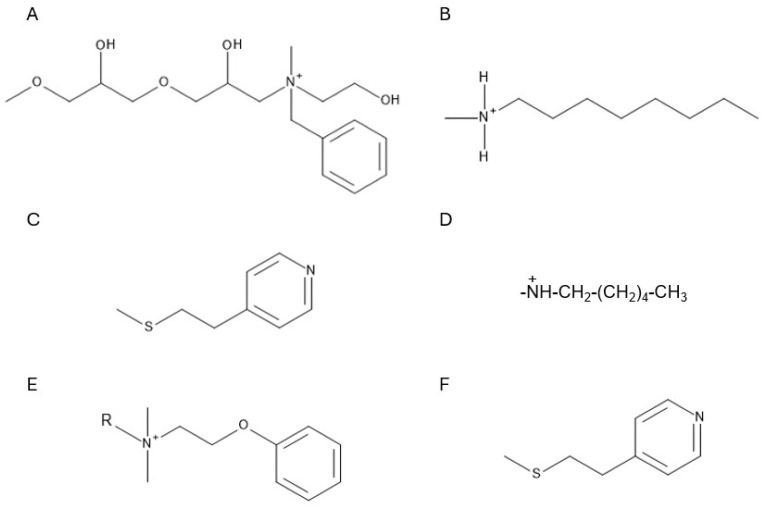
Chemical structures of the Capto Adhere (**A**), Capto Core 700 (**B**), PPA HyperCel (**C**), HEA HyperCel (**D**), Nuvia aPrime 4A (**E**) and MEP HyperCel (**F**) ligands.

Multimodal anion exchangers, particularly resin bead particles, are among the most effective multimodal adsorbents for antibody aggregate separation. Widely used commercial resins include Capto Adhere [134], Capto Adhere ImpRes [134], and Capto Core Series [135] (Cytiva, Marlborough, MA, USA), as well as HEA Hypercel [136], PPA Hypercel [137,138] (Sartorius, Göttingen, Germany), and Nuvia aPrime 4A [31,32] (Bio-Rad, Hercules, CA, USA). These strong multimodal ion-exchange resins contain ligands with primary amine groups, enabling ion-exchange interactions, while aromatic or aliphatic groups provide hydrophobic interactions. Molecular structures of these ligands are depicted in Figure 4.

The most well-known weak multimodal anion exchanger is MEP HyperCel (Sartorius, Göttingen, Germany) which was initially promoted as an adsorbent for hydrophobic charge induction chromatography (HCIC) [139]. A key advantage of HCIC is that it enables protein separation under mild adsorption and elution conditions, achieving a high degree of separation through a step pH change [139]. MEP HyperCel features a 4-mercaptoethylpyridine (MEP) ligand with a pKa of 4.8. Its structure is shown in Figure 4F. Proteins bind to this ligand primarily through hydrophobic interactions, supplemented by thiophilic binding at near-neutral pH. For elution, a pH shift to 4–4.5 is used [140,141]. MEP HyperCel was developed as a potential alternative to protein A resins [139]. Consequently, early studies focused on comparing its performance with various protein A adsorbents [129,142,143]. However, the high ProA specificity for target proteins outweighs the advantages offered by MEP HyperCel.

MEP HyperCel was also investigated early on, alongside Capto Adhere and several hydroxyapatite resins, for the separation of antibody aggregates [125,144]. In both cited studies, MEP HyperCel demonstrated the highest overall efficiency in removing high-molecular-weight (HMW) aggregates. However, Capto Adhere achieved superior dimer removal and exhibited significant capacity for host cell protein (HCP) binding [125].

Moreover, in a separate study, Capto Adhere outperformed conventional single-mode ion exchangers and hydrophobic adsorbents in removing heterogeneously charged aggregates from post-protein A CHO supernatant [145]. In this study, a successful scale-up by a factor of more than 1700 was achieved, along with a 92% antibody yield and an 85% reduction in aggregates, using the design of experiments (DoE) method. Additionally, Capto Adhere demonstrated the highest purity (99.6%) and yield (92%) in a comprehensive study comparing eight conventional and mixed-mode adsorbents that achieved a comparable level of aggregate removal [130]. The conditions and results of this study for Capto Adhere are in Table 1.

Capto Adhere ImpRes, which is a variant of Capto Adhere with an average bead size (36–40 μm compared with 75 μm in the conventional Capto Adhere) was found to be equally effective in reducing aggregate content [146,147]. Because protein interactions with multimodal adsorbents are influenced by the presence of salts, the effect of kosmotropic salts on the separation efficiency of antibody aggregates was investigated for Capto Adhere, Capto Adhere ImpRes, and Nuvia aPrime 4A [131]. Results for Nuvia aPrime 4A are summarised in Table 1. This resin has been investigated in several studies focusing on multicomponent adsorption properties, BSA separation, adsorption-induced aggregation, and protein selectivity [31,32,148].

The performances of HEA Hypercel and PPA Hypercel were studied by Pezzini et al. [133,138], who examined the effects of pH on the dynamic binding capacity of various proteins, as well as the impact of additives and media conductivity on mixed-mode interactions. They also compared their performance with that of several other monomodal and multimodal adsorbents [133,138]. In two additional studies, PPA Hypercel and HEA Hypercel were evaluated alongside Capto Adhere for the removal of mAb fragments [149] and for the separation of antibody charge variants and aggregates [132]. Results from aggregate removal experiments with HEA Hypercel are presented in Table 1.

An interesting application of multimodal anion exchangers was demonstrated in the study by Maria et al. [150], where a three-step purification procedure was developed for mAb separation from a cell culture supernatant. HEA Hypercel was utilized in the initial capture step, followed by the multimodal cation exchangers Capto MMC and Capto MMC ImpRes for intermediate purification. The final polishing step was performed using two membrane adsorbents: the strong anion exchanger Sartobind Q and the multimodal anion exchanger Sartobind STIC. The optimized purification process achieved an 88% yield and 99.9% purity [150].

Sartobind STIC has also been used for HCP clearance [151] and for the separation of model macromolecules, BSA and salmon sperm DNA [83]. Another commercially available multimodal anion-exchange membrane is Purexa MQ (Donaldson), which was the focus of a study by Osuofa et al. [152]. Their work explored the use of Purexa MQ for protein polishing, evaluating its dynamic binding capacities for IgG, BSA, and salmon sperm DNA under varying conductivity and pH conditions. Additionally, the study examined the membrane’s effectiveness in monoclonal antibody (mAb) clearance from HCP and aggregates.

##### Multimodal Cation Exchangers

**Figure 5:**
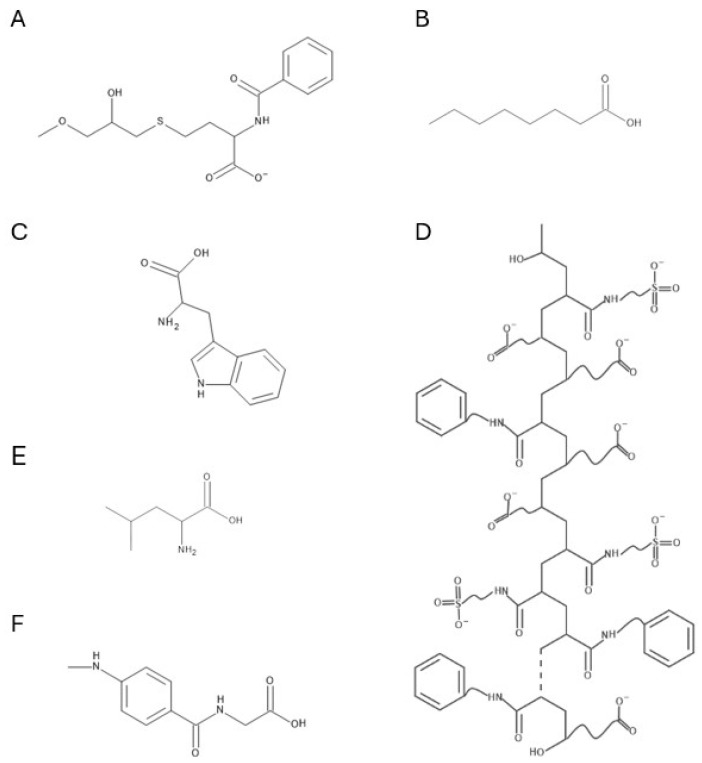
Chemical structures of the Capto MMC (**A**), Poros Caprylate (**B**), Toyopearl MX-Trp-650M (**C**), Eshmuno HCX (**D**), Eshmuno CMX (**E**), and Nuvia cPrime (**F**) ligands.

In the field of mAb purification, the most frequently reported multimodal cation exchanger is Capto MMC (Cytiva, Marlborough, MA, USA). This features a carboxylic group, classifying it as a weak cation exchanger. Its structure, which is shown in Figure 5A, also contains a benzene ring providing hydrophobic interaction, an amine group serving for hydrogen bonds and sulphur for thiophilic interactions. The properties of Capto MMC are often compared with Capto Adhere. Song et al. employed these two adsorbents for the separation of aggregates and fragments of bispecific antibodies (bAb) [153]. In another study, they were tested for the separation of the isoforms of model proteins, including BSA and Fc-fusion etanercept [154]. Tang et al. investigated Capto MMC ImpRes, a higher efficiency variant, for the removal of impurities associated with bispecific antibody production [155].

Capto MMC has been explored in various studies as an option for antibody capture from cell culture media [156,157]. Results from the testing of Capto MMC for the capture step can be found in Table 2. In the work of Joucla et al., Capto MMC was compared with a conventional cation exchanger for its ability to capture antibodies from CHO supernatants [158]. Additionally, Capto MMC has been used to capture antibody fragments across a broad range of pH and conductivity conditions [159]. To highlight the advantage of high salt tolerance in multimodal resins, Zhang et al. utilized Capto MMC and hydroxyapatite for antibody separation from a high-salt post-protein A fraction. These resins effectively removed protein aggregates and residual protein A, achieving up to 97% antibody purity [160].

Other multimodal weak cation exchangers are Poros Caprylate (ThermoFisher Scientific, Waltham, MA, USA), Toyopearl MX-Trp-650M (Tosoh Biosciences, San Francisco, CA, USA), Diamond MMC Mustang (Bestchrom, Shanghai, China), Eshmuno HCX and Eshmuno CMX (both Merck Millipore, Burlington, MA, USA), and Nuvia cPrime (Bio-Rad, Hercules, CA, USA). Their chemical structures are shown in Figure 5. Vajda et al. investigated the impact of dual salt mixtures on mAb aggregate removal using Toyopearl MX-Trp-650M, demonstrating its potential in optimizing aggregate clearance [161]. Their results are summarised in Table 2.

Meanwhile, Zhang et al. have reported that Diamond MMC exhibits a slightly higher dynamic binding capacity than Capto MMC ImpRes, while also providing comparable resolution under identical conditions [162]. The purity and yield reached with Diamond MMC Mustang for bispecific antibody are presented in Table 2.

Eshmuno HCX and Eshmuno CMX feature ligand tentacles that create a three-dimensional structure, enhancing protein accessibility and improving separation efficiency. These resins have been evaluated alongside Capto MMC and Nuvia cPrime for the purification of highly hydrophobic complex antibody formats [164]. Using Eshmuno CMX, a purity of over 97% and a recovery rate of approximately 70% were achieved. Beyond purification, Eshmuno HCX has also been investigated by Wang et al. for the direct capture of IgG from undiluted cell culture broth [163]. The results can be found in Table 2.

He et al. investigated the use of Nuvia cPrime for the removal of high-molecular-weight (HMW) aggregates from an IgM pool. When used in series with a second column packed with CHT resin, the process successfully yielded an HMW-free product [166]. Yan et al. focused on the adsorption behaviour and separation efficiency of Nuvia cPrime for immunoglobulin and bovine serum albumin [167]. Robinson et al. explored the impact of pH on antibody retention using Nuvia cPrime alongside Capto MMC [168].

Additionally, DiLeo et al. assessed the potential of Capto MMC, Toyopearl MX-Trp-650, MEP HyperCel, and Nuvia cPrime for antibody capture [165]. Their findings indicate that these multimodal cation exchangers achieved binding capacities comparable to those of protein A resins, without requiring extensive feedstock manipulation. Moreover, using multimodal adsorbents in the capture step reduced the likelihood of aggregate formation. The concept of multimodal capture was also the focus of a recent study by Arakawa [169].

### 4.3. Non-Commercial Multimodal Adsorbents

The successful implementation of commercial multimodal resins in purification processes has driven further research into the development and testing of new adsorbent prototypes. Gao et al. used two custom-made resins featuring benzylamine and butylamine ligands for the separation of IgG aggregates [134]. Their performance was compared with Capto Adhere, a well-established multimodal ion exchanger. Additionally, the study evaluated Q Sepharose FF (traditional ion-exchanger) and Phenyl Sepharose 6 FF (a hydrophobic interaction resin) to assess their individual contributions to mixed-mode interactions.

Gu et al. developed and tested three dextran-grafted resins functionalized with a benzylamine ligand to evaluate their adsorption capacity and salt tolerance for BSA and bovine IgG [170]. These modified resins exhibited significantly improved binding capacities for both proteins compared with their non-grafted counterparts. In a further study, Gu et al. designed mixed-mode adsorbents incorporating tailored ligand functional groups, specifically 4-(aminomethyl)phenol (AMP) and 4-(aminomethyl)benzonic acid (AMA) [171]. The adsorption performance of these adsorbents was assessed using IgG and BSA as model proteins.

Liu et al. introduced a novel adsorbent by integrating 5-aminobenzimidazole and 3-aminophenylboronic acid into an agarose matrix modified by triazine [172]. Protein binding on these ligands occurred through a combination of boronate affinity and hydrophobic charge induction, enabling a two-site adsorption mechanism. This advanced adsorbent was successfully tested for the separation of IgG from human serum and mAb from cultivation media [172].

Aoyama et al. developed novel hydrophobic multimodal adsorbents by modifying polyallylamine (PAA) with butyl and phenyl functional groups [173]. Their comprehensive study evaluated these resins for mAb purification, specifically targeting HCP and aggregates. The study also benchmarked their performance against the widely used Capto Adhere.

LeBarre et. al. combined the principles of size-exclusion chromatography and mixed-mode ligand interactions to develop an innovative mixed-mode size-exclusion resin [174]. Their work explored various ligand chemistries, demonstrating the resin’s effectiveness in separating antibody aggregates and fragments.

Kanwar Shekhawat et al. systematically expanded the landscape of multimodal chromatography by chemically modifying Capto MMC to create a diverse database of novel resins [175]. They synthesized representative samples and rigorously tested their selectivity for the separation of high molecular weight aggregates, providing valuable insights into the design of next-generation multimodal adsorbents.

Liu et al. conducted an extensive study aimed at enhancing the performance of HCIC resins through dextran grafting [176]. Building on the research of Xia et al. [177] and Gao et al. [178], they developed a novel resin by incorporating 2-mercapto-1-methylimidazole as the HCIC ligand into dextran-grafted agarose. Their work systematically examined the influence of ligand density, salt concentration, and pH on immunoglobulin adsorption, providing critical insights into resin optimization [179].

Lu et al. further explored the application of 2-mercapto-1-methylimidazole as an HCIC ligand by investigating its adsorption properties on resins with varying pore sizes and ligand densities [180]. Their study provided a comprehensive analysis of IgG adsorption isotherms and kinetics, offering valuable insights into optimizing resin design for enhanced protein purification efficiency.

Building on advancements in polymer-modified HCIC resins, researchers have also focused on the synthesis and characterization of polymer-grafted materials via atom transfer radical polymerization [181,182]. Within this research initiative, Fang et al. developed two innovative resins—PEI-4FF and Ac-YFRH-PEI-4FF—by grafting poly(ethylenimine) onto a highly cross-linked agarose matrix [183]. To assess their separation performance, adsorption isotherms of BSA and IgG were measured, demonstrating their potential for improved protein binding and purification.

Tong et al. advanced the field of HCIC by designing a novel ligand, tryptophan-5-amino-benzimidazole (W-ABI), aimed at improving both specificity and protein binding efficiency [184]. In this ligand, the tryptophan functional group enhances affinity for the IgG constant region, while 5-amino-benzimidazole facilitates efficient protein elution. Their study evaluated the adsorption equilibrium, dynamic binding capacity, and effectiveness of IgG separation from protein mixtures and cell culture supernatants [184].

With the continuous rise in upstream productivity, downstream processing must adapt to meet increasing capacity demands. One promising solution is the use of chromatographic membranes, which offer high flow rates and scalability. Wang et al. developed a multimodal cation-exchange membrane via graft polymerization for antibody purification [185]. They extensively characterized the membrane, analysing its static binding capacity across varying pH and ionic strength. In a subsequent study, a similar membrane was tested under dynamic conditions, where its performance was benchmarked against commercial multimodal adsorbents [186].

Fan et al. took a different approach by designing a nonwoven multimodal cation-exchange membrane, which demonstrated superior removal of HCP and aggregates when compared with Capto MMC and Toyopearl MX-Trp-650M [187]. Similarly, Ma et al. explored surface modification to develop a HCIC membrane, which was intended to enable IgG separation from BSA [188].

The latest advancement in this field has been made by Pham et. al., who introduced a novel multimodal membrane functionalized with 2-mercapto-3-carboxylic acid as a ligand [189]. Their membrane exhibited strong purification capabilities for variable antibody fragments from cell culture media [189].

## 5. Concluding Remarks

As the biopharmaceutical industry advances, the purification of monoclonal antibodies remains a critical aspect of ensuring product quality and regulatory approval. The presence of antibody aggregates poses significant challenges, necessitating robust separation strategies that balance efficiency, selectivity, and scalability. Multimodal chromatography has emerged as a powerful tool in aggregate removal, leveraging the synergistic effects of multiple interaction modes to enhance purification performance.

This review highlights the growing role of multimodal chromatography as a complement or alternative to traditional single-mode techniques. The assessment of commercially available resins, as well as novel prototype adsorbents and membranes, underscores the ongoing efforts to refine purification technologies for improved aggregate clearance and process adaptability. Despite its advantages, multimodal chromatography still presents challenges, including resin optimization, process integration, and cost considerations, which warrant further investigation.

Future research should focus on developing resins with enhanced binding specificity, optimizing operational parameters for large-scale manufacturing, and integrating multimodal strategies with other purification techniques to maximize overall process efficiency. As therapeutic antibody production continues to scale up, advancements in chromatographic purification will be essential to meeting stringent industry standards while maintaining high yields and cost-effectiveness.

## Figures and Tables

**Table 2:** The most commonly used multimodal CEX resins and selected applications.

Adsorbent	Operational Mode	Position	Product	Reference
Capto MMC	Bind-elute	Capture step	Monomeric IgG with >99% purity and 82% yield	[157]
Toyopearl MX-Trp-650M	Bind-elute	Polishing step	Monomeric mAb with 90% yield and aggregate removal factor 6.5 (from original 15%)	[161]
Diamond MMC	Bind-elute	Polishing step	Monomeric bAb with 98.8% purity and 55.2% yield	[162]
Eshmuno HCX	Bind-elute	Capture step	Monomeric IgG with purity > 99.5% and recovery 94.6%	[163]
Eshmuno CMX	Bind-elute	Polishing step	Monomeric IgG with >97% purity and 70% recovery	[164]
Nuvia cPrime	Flowthrough	Capture step	Dynamic binding capacity 55 mg/mL	[165]
